# Social, health-related, and environmental factors influencing sleep problems of children, adolescents and young adults

**DOI:** 10.25646/9879

**Published:** 2022-06-08

**Authors:** Petra Kolip, Ronny Kuhnert, Anke-Christine Saß

**Affiliations:** 1 Bielefeld University, School of Public Health; 2 Robert Koch Institute, Berlin Department of Epidemiology and Health Monitoring

**Keywords:** SLEEP PROBLEMS, CHILDREN, ADOLESCENTS, YOUNG ADULTS, SOCIAL CONDITIONS, ENVIRONMENTAL FACTORS, NOISE, KIGGS

## Abstract

Sleep is a relevant factor for functioning and well-being of young people. The paper provides a differentiated description of sleep difficulties in this population group including social, health-related, and environmental factors. The analyses included n=6,728 11- to 17-year-olds of the KiGGS baseline study (2003–2006) and 6,072 young adults (age 18–31), who provided information relating sleep in the survey KiGGS Wave 2 (2014–2017). Information from 3,567 people was evaluated at two survey points. 22.0% of the 11- to 17-year-olds reported sleep difficulties. A significant impact for the sex (female), living with a single parent, and with siblings is reflected in the logistic regression. The risk for sleep difficulties increases significantly in the case of mental problems and pain. Among the 18- to 31-year-olds, 19.6% complained of difficulties falling asleep and sleeping through the night. In addition to sex, noise exposure, a low level of education, the professional situation, and living with children were reflected as important influencing factors in the logistic regressions. Over one third of those, who suffered from sleep problems as children and adolescents, also indicated sleep difficulties almost ten years later. The high prevalence of sleep problems and the associated health risks illustrate the high public health relevance of the topic. In addition to sex, health-related and environmental variables also turned out to be significant and need to be considered in the development of interventions.

## 1. Introduction

Sleep is a relevant factor for the healthy development of children and adolescents and, due to the regenerative processes associated with it, is central for health and well-being. Sufficient and undisturbed sleep is essential for cognitive functioning and performance [1–3] and is positively associated with academic performance in children and adolescents (for an overview, see [3–5]). Physical parameters, such as, for example, overweight/obesity and cardiovascular risk factors (e.g. elevated blood lipid level and high blood pressure) affect sleep duration and sleep quality [6–12]. Similar findings are reflected for the connection between sleep problems and aspects of mental health [4, 13, 14]. For instance, associations with hyperkinetic disorders, conduct disorders, anxiety disorders, and depression can be found.

Comparable patterns are reflected in adulthood. For instance, insomnia, thus difficulties falling asleep and sleeping through the night, which occur frequently and which are associated with poor sleep quality as well as adverse effects during the day, are considered to be risk factors for cardiovascular diseases [15, 16], diabetes mellitus [17], and accidents [18]. They additionally increase the probability of mental disorders, in particular depression, anxiety disorders, and addictions [19–22].

It has been widely proven that sleep problems in school-aged children are common. A review reports a range from 9% to 42% for insufficient sleep [23]. Analyses of the baseline study of the German Health Interview and Examination Survey for Children and Adolescents (KiGGS) [24] show that 23.1% of the 11- to 13-year-olds and 20.7% of the 14- to 17-year-olds are affected by sleep difficulties, girls more frequently than boys (in the age group of 14- to 17-year-olds, this difference also reaches statistical significance). Information relating to the further social differentiation is not yet available for the KiGGS baseline study. International surveys support a social gradient for sleep problems, and children from socially disadvantaged families are also affected more strongly by the negative effects of a lack of sleep than children from socially advantaged families [3, 25].

Information relating to the prevalence of sleep problems of adults is available from the German Health Interview and Examination Survey for Adults (DEGS1, 2008–2011). It shows that 10.4% of the 18- to 39-year-old women and 7.6% of the men of the same age are affected by difficulties falling asleep three or more times per week, 17.9% of the women and 9.5% of the men are affected by difficulties sleeping through the night [26]. 24.1% of the women and 17.8% of the men of this age group report a poor sleep quality, which leads to a use of sleeping pills among 1.9% of the women and 0.8% of the men at least once per week. Differentiated analyses, focussing on young adulthood, which allow making a further social differentiation, including social, health-related, and environmental factors, are not yet available.

The causes of sleep disorders are diverse. Following a socioecological model, good sleep is influenced by personal factors (e.g. behaviour, preferences in sleep-wakefulness cycle, comorbidities) as well as by (inter-)personal, social, and societal factors [22]. At the environmental level, an important influence on sleep is attributed [28] to noise and light [27] and psychosocial stressors. In addition, socially disadvantaged groups of people are more frequently affected by factors, which are associated with a poorer sleep quality [26, 29–33].

Sleep disorders are said to be highly chronic [34, 35], but information relating to the percentage of young adults with sleep disorders, who already suffered from sleep difficulties during adolescence, is not yet available for Germany.

With its baseline study from 2003 to 2006, KiGGS provided representative data relating to the sleep of children and adolescents for the first time [24]. This set of data allows for a differentiated evaluation in order to identify factors influencing sleep difficulties. About ten years later, the participants of the KiGGS baseline study were surveyed again [36, 37], so that a differentiated evaluation can also be made for young adults, and data relating to the stability of sleep difficulties in the transition from adolescence to young adulthood are available as well.

The following questions are thus to be discussed in this paper:

1. How common are sleep difficulties in childhood, adolescence, and young adulthood, and how does the prevalence of sleep problems differentiate along sociodemographic features?
2. What influence do sociodemographic variables (age, sex, socioeconomic status of the family, migration background, type of family, professional status) as well as health-related (mental health, pain) and environmental factors (place of residence, noise) have?
3. How high is the percentage of children and adolescents, among which sleep difficulties were reported in the KiGGS baseline study as well as in young adulthood in KiGGS Wave 2?

**The KiGGS study**

The German Health Interview and Examination Survey for Children and Adolescents

**Data owner:** Robert Koch Institute

**Aim:** Providing reliable information on health status, health-related behaviour, living conditions, protective and risk factors, and health care among children, adolescents and young adults living in Germany, with the possibility of trend and longitudinal analyses

**Study design**: Combined cross-sectional and cohort study

**KiGGS survey waves:**

▶ KiGGS baseline study (2003–2006) interview and examination survey
▶ KiGGS Wave 1 (2009–2012) interview survey
▶ KiGGS Wave 2 (2014–2017) interview and examination survey

**KiGGS cross-sectional study**

**Population:** Children and adolescents with permanent residence in Germany

**Age range:** 0–17 years

**KiGGS cohort study**

**Sampling:** Re-invitation of everyone who took part in the KiGGS baseline study (n=17,641) and who was willing to participate in a follow-up

**Age range KiGGS Wave 1:**

6–24 years (n=11,992)

**Age range KiGGS Wave 2:**

10–31 years (n=10,853)

More information is available at www.kiggs-studie.de/english

## 2. Methods

### 2.1 Base data: The KiGGS study

The KiGGS baseline study (2003–2006) included a total of n=17,640 children and adolescents between the ages of 0 and 17. Due to the fact that self-reported and proxy information relating to the sleeping behaviour can differ [38], this paper focusses on the group of the 11- to 17-year-olds, for whom self-reported information relating to difficulties falling asleep and sleeping through the night are available (n=6,728). Questions relating to the topic of sleep were not asked in KiGGS Wave 1, a first follow-up survey from 2009 to 2012, but were asked in the second follow-up survey (KiGGS Wave 2, 2014–2017). All former survey participants of the KiGGS baseline study were invited to KiGGS Wave 2, unless they had moved abroad or could not be contacted any longer for KiGGS Wave 1 [36]. The data from 6,072 young adults (18–31 of age) was included in the analyses. Self-reported information relating to the sleep quality was available from 3,567 people at two survey points. At the time of the KiGGS baseline study, they were between the ages of 11 and 17 and between 21 and 31 at the time of the second survey.

### 2.2 Operationalisation of the variables

#### Sleep difficulties

The 11- to 17-year-old children and adolescents answered the question whether they suffer from sleep difficulties (answer format: yes/no). A differentiation was then made according to difficulties falling asleep and sleeping through the night; this information was not used in the following analyses. Difficulties falling asleep and sleeping through the night in the last four weeks were assessed in KiGGS Wave 2. In contrast to the KiGGS baseline study, the answer format was more differentiated: ‘Not at all /fewer than once per week/once or twice per week/three times per week or more frequently per week’. Analogously to the paper by Schlack et al. [26], sleep difficulties were encoded when problems falling asleep or sleeping through the night occurred at least three times per week.

#### Education

The respondents or their parents, respectively, provided information relating to their highest completed level of education. To classify the information, the International Standard Classification of Education (ISCED) was used [39]. ISCED takes into account academic as well as professional levels of education. The completed level of education of the parents was used in the KiGGS baseline study (maximum value for each household). The young adults were asked themselves in KiGGS Wave 2.

#### Vocational training-/occupation-related living situation

Questions about the current living situation were asked in eleven categories in KiGGS Wave 2. For the analyses, they were combined into three groups: Full-time employees (incl. military service, voluntary social year), part-time employees (incl. mini job, pupils, students, and trainees), unpaid work/no employment (e.g. parental leave, homemaker, unemployed, unable to work).

#### Migration background

Children and adolescents born in Germany and who have one parent who immigrated from another country and/or who does not have German citizenship, are considered to be participants with one-sided migration background in the KiGGS study. Children and adolescents, for whom this applies to both parents, have a two-sided migration background [40]. When the participants themselves have immigrated from another country and at least one parent was not born in Germany, they are assigned to the group with two-sided migration background.

#### Type of family

The type of family captures two aspects in the KiGGS baseline study: Firstly, parents were asked whether children and adolescents are growing up with one or with both parents. Secondly, information was provided as to whether siblings also live in the household.

KiGGS Wave 2 was interested in whether the respondents live with a partner in the same household (yes/no). In addition, it was asked whether the respondents live in the household with children. It was possible to deduce from the free text information relating to all household members, whether they are younger siblings or whether they are children or stepchildren, respectively. People with (step)children in the household were compared to those without (step)children in the analyses.

#### Place of residence

On the basis of the information relating to the place of residence (municipal code), a classification by political municipal size class was made. The following categories were differentiated: rural (<5,000 residents), provincial (5,000 to <20,000 residents), urban (20,000 to <100,000 residents), and metropolitan (≥100,000 residents). For the regression analyses, the characteristic values of the variables were combined into two categories: rural versus other places of residence.

#### Noise exposure

Only KiGGS Wave 2 asked about the noise exposure in the flat/in the house (last twelve months). Eleven noise sources were specified (e.g. street noise, noise by restaurants, noise by family members). The participants were able to indicate their exposure for each noise source on a five-point scale. For the analyses, all participants, who had indicated a high or very high exposure in the case of at least one noise source, were assigned to the category ‘noise exposure’.

#### Pain

The children and adolescents in the KiGGS baseline study indicated whether they had pain in the past three months (yes/no). Such information is not available for KiGGS Wave 2.

#### Mental health

To assess mental problems, the Strengths and Difficulties Questionnaire (SDQ) [41] was used in the KiGGS baseline study, which captures four areas of concern (emotional problems, behavioural problems, hyperactivity problems, and problems in dealing with peers), which are aggregated to a total score. This permits a classification of the results into the ranges ‘abnormal’, ‘borderline’, and ‘normal’. For the regression analyses, the characteristic values of the variables were combined into two categories: Normal versus borderline or abnormal scores.

KiGGS Wave 2 focussed on depressive symptoms among the adult participants. These symptoms were captured by means of the Patient Health Questionnaire (PHQ-9), which captures nine symptoms during the past two weeks using a four-step response format (from ‘not at all’ to ‘almost daily’). A moderate to severe symptomatology is present in the case of scores above 10, a mild one in the case of scores between 5 and 9 [42]. For the regression analyses, the characteristic values of the variables were combined into two categories: no depressive symptoms versus mild, moderate, or severe symptoms.

### 2.3 Statistical methods

To answer the first question, the information from the participants of the KiGGS baseline study (aged 11–17) and KiGGS Wave 2 (aged 18–31) were evaluated descriptively and examined for group differences using the chi-square test. In the next step, the association between several variables and sleep problems is estimated based on odds ratios by means of an adjusted logistic regression. The odds ratio indicates the factor by which the statistical ‘chance’ of a health impairment (here: reporting sleep problems) is increased in one group compared to a reference group. Sociodemographic and environmental variables were included in Model 1. Mental health was additionally considered in Model 2 (SDQ total score among children and adolescents, and depressive symptoms (PHQ-9) among the young adults). Among children and adolescents, the question about pain was furthermore added. For the third question, the information of those, who participated in the survey twice, was analysed. Information relating to sleep problems at two points in time at an interval of approximately ten years was available from 3,567 people. Transitions are shown descriptively.

Adjustment factors, which compensate for possible distortions of the sample due to selective repeated participation, were used in the statistical analyses [36, 43]. SAS (version 9.4) and R (version 3.6.1) were used to analyse the data, using survey procedures in order to take into account the survey design and the adjustment. p-values of less than 0.05 are considered to be statistically significant.

## 3. Results

###  

#### Sleep problems in childhood and adolescence

3,287 girls and 3,441 boys between the ages of 11 and 17 answered the question on sleep difficulties in the KiGGS baseline study (2003–2006). The percentage of girls in the sample is 48.9%. At the time of the baseline study, 6.6% of children and adolescents lived in a family, which is assigned to the low education group, 48.0% in a family with medium, and 45.3% with high level of education. 5.7% of children and adolescents had a one-sided, 15.2% a two-sided migration background. 12.5% of participants lived with only one parent, 73.9% lived with at least one sibling in the household. 21.3% of children and adolescents lived in rural municipalities, 26.0% in provincial, 29.5% in urban, and 23.2% in metropolitan municipalities.

The prevalence of sleep difficulties in this group depending on sociodemographic and health variables can be seen in Table 1. It is noteworthy that the prevalence of sleep difficulties varies significantly with sex and living conditions of the children and adolescents (Table 1). Almost one quarter of the girls (24.9%) reported sleep difficulties, and 19.2% of the boys. In families with a single parent, 27.9% of participants reported sleep difficulties, versus 21.0% of children and adolescents from couple households. For other sociodemographic variables, such as, age of participants, education of parents, a migration background, living with siblings, or the size of the place of residence, no significant differences related to sleep quality were found.

Experiencing sleep difficulties additionally varied with pain conditions. 24.5% of children and adolescents, who had pain in the last three months, reported difficulties falling asleep and sleeping through the night (participants without pain conditions: 13.2%). The risk for mental and behavioural problems was also associated with sleep problems: Children and adolescents with a borderline (42.4%) or abnormal (47.9%) SDQ total score reported sleep problems significantly more frequently than girls and boys with normal SDQ total score (18.7%).

**Figure 1:**
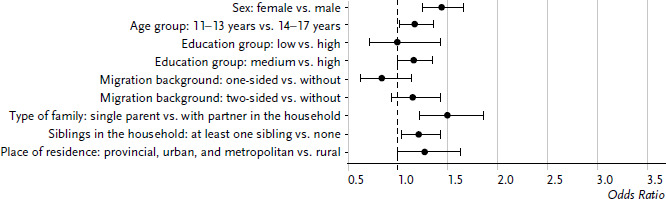
Odds ratios for sleep problems among 11- to 17-year-old girls and boys according to sociodemographic variables (n=3,287 girls, n=3,441 boys) Source: KiGGS baseline study (2003–2006)

In a second step, these variables were included in a logistic regression, the characteristic values of the variables were partly grouped for this purpose (see chapter 2.2 Operationalisation of the variables). In Figure 1, odds ratios are plotted, thus probabilities for reporting difficulties falling asleep or sleeping through the night. It became apparent that growing up in the household of a single parent was associated with the largest risk for a poor sleep quality (OR=1.50, 95% CI: 1.21–1.87). Sex ranks second: Among girls, compared to boys, the risk for sleep difficulties was increased significantly by the factor 1.44 (95% CI: 1.24–1.67). Living together with siblings ranks third. With siblings in the household, the risk for poor sleep quality was likewise increased (OR=1.21, 95% CI: 1.03–1.43). A slight, but marginally significant influence became apparent in the case of age. Additional features, such as migration background, education group of the parents, or the place of residence (rural or urban) had no influence in this analysis. When performing the analyses separately for girls and boys, it became apparent that among the girls, none of the included variables is significantly associated with the self-reported sleep quality. Among the boys, significantly increased odds ratios resulted for poor sleep quality among those, who grew up in the household of a single parent, lived with siblings, and were in the younger age group (aged 11–13).

**Figure 2:**
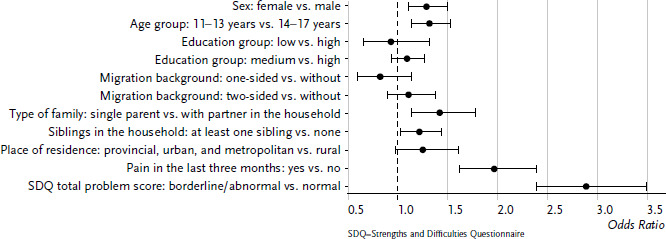
Odds ratios for sleep problems among 11- to 17-year-old girls and boys according to sociodemographic and health variables (n=3,287 girls, n=3,441 boys) Source: KiGGS baseline study (2003–2006)

Two variables relating to health (mental abnormalities and pain, Figure 2) were additionally included in a second regression model. It becomes apparent that the risk of reporting sleep problems is significantly higher when the children and adolescents were affected by mental problems (SDQ total problem score borderline or abnormal OR=2.89, 95% CI: 2.39–3.49) or pain (OR=1.97, 95% CI: 1.62–2,39). When performing the analyses separately for girls and boys, the large influence of the health variables on sleep quality is confirmed. Odds ratios for sleep difficulties in the case of mental problems were even slightly higher among girls than among boys.

#### Sleep problems in young adulthood

3,384 women and 2,688 men between the ages of 18 and 31 answered the question on sleep difficulties in KiGGS Wave 2 (2014–2017). All participants had already participated in the baseline study, some had answered the sleep questions themselves (11- to 17-year-olds). The percentage of the women in this group is 55.7%. 15.1% of the participants were assigned to the low education group at the time of the follow-up survey, 63.8% to the medium, and 21.1% to the high education group. In response to the question relating to the vocational training or occupation-related living situation, 49.8% of the respondents indicated a full-time employment. 43.3% were employed part-time, and 6.9% performed unpaid work or were not employed at the time of the survey. 6.1% of the young women and men had a one-sided, 10.5% had a two-sided migration background. 27.2% of the respondents lived with a partner, 6.2% with (step)children in the household. 15.7% of the respondents lived in rural, 25.1% in provincial, 26.4% in urban, and 32.8% in metropolitan municipalities.

The result pattern already described for children and adolescents was also reflected in young adulthood (Table 2): Young women reported sleep difficulties significantly more frequently than young men (26.2% vs. 13.3%). The level of education was also relevant: With a prevalence of 14.2%, young adults from the high education group had sleep problems significantly more rarely than people with medium (20.0%) or low education level (23.5%). As already among the 11- to 17-year-olds, a migration background and the age also did not play a role among the 18- to 31-year-olds. Living with a partner was likewise not relevant but living with children was: 35.2% of those, who lived with children in the household, reported difficulties falling asleep or sleeping through the night, compared to 17.9% young adults in childless households. A sex-differentiated analysis showed a significant difference for women, not for men. However, this could also be related to the small group size, because only a small portion of men between the ages of 18 and 31 lived with children in the household and the confidence interval is correspondingly large. There were no significant differences for the size of the place of residence in relation to sleep quality.

Noise exposure predictably had a significant effect: 31.3% of those suffering from noise exposure indicated being affected by sleep problems. In the group of those, who were not affected by noise, this number was only 16.5%. A particularly pronounced effect became apparent for depressive symptoms: More than half of the young adults with moderate to severe symptoms (50.6%) suffered from sleep problems, compared to 23.8% with mild, and 6.2% without symptoms.

**Figure 3:**
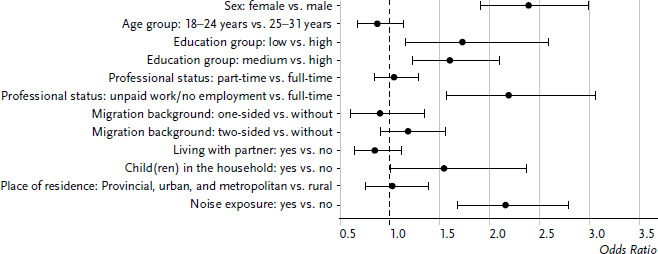
Odds ratios for sleep problems among 18- to 31-year-old women and men according to sociodemographic variables (n=3,384 women, n=2,688 men) Source: KiGGS Wave 2 (2014–2017)

In a second step, these variables were included in a logistic regression. The characteristic values of the variables were partly grouped before they became part of the model (see chapter 2.2 Operationalisation of the variables). Odds ratios and confidence intervals are plotted in Figure 3. Compared to children and adolescents, significant connections to the sleep quality resulted among young adults in the case of significantly more variables. Sex had the strongest connection: Compared to men, women had more than twice the risk of reporting sleep difficulties (OR=2.39, 95% CI: 1.91–2.99). Participants with high or very high noise exposure also reported sleep problems more than twice as frequently as people, who are not affected by noise (OR=2.16, 95% CI: 1.67–2.79). A significantly increased risk was also determined for young adults with low education level compared to high education level (OR=1.73, 95% CI: 1.15–2.59). Additional significant influencing factors were the working conditions (full-time vs. no employment, with a higher risk for people without employment) and children in the household (with a higher risk when living with children). When performing the analyses separately for women and men, there are partly shifts in the significance of the influencing factors (data not shown). Among women, the influence of education on sleep quality was highest, followed by noise exposure and living situation. Hardly any significant connections followed among men: Only noise exposure was connected with sleep quality.

**Figure 4:**
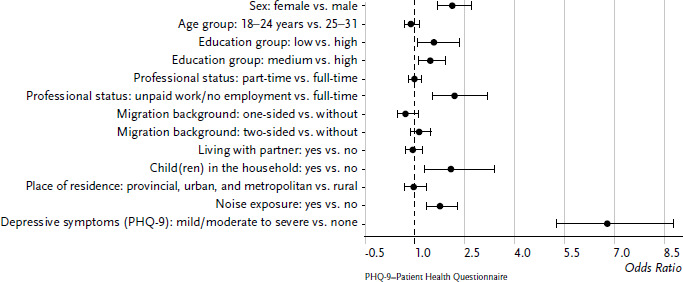
Odds ratios for sleep problems among 18- to 31-year-old women and men according to sociodemographic variables and mental health (n=3,384 women, n=2,688 men) Source: KiGGS Wave 2 (2014–2017)

A variable relating to mental health was additionally included in a second regression model: Mild to severe depressive symptoms according to PHQ-9 (Figure 4). It became clear that the risk of sleep difficulties is significantly higher when the young adults report depressive symptoms in the last two weeks (OR=6.79, 95% CI: 5.25–8.79). When performing the analyses separately for women and men, the large influence of mental health on the sleep quality is confirmed. The odds ratios for sleep difficulties in the case of mental problems were even slightly larger among men than among women (data not shown).

Due to the fact that the participants in the KiGGS baseline study were asked to participate in the KiGGS study in the long term, it was possible to combine information relating to the sleep behaviour in childhood or adolescence, respectively, with the information in young adulthood from 3,567 participants. Those, who had answered the questions on sleep quality at both survey points, thus were at least eleven years old during the KiGGS baseline study, were included in the following analysis.

**Figure 5:**
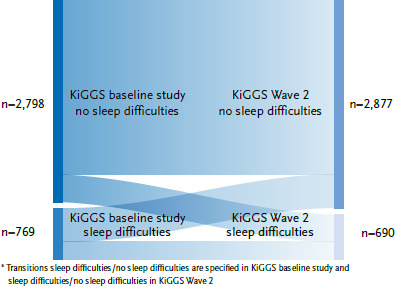
Prevalence of sleep problems at two survey points (11- to 17-year-olds and 21- to 31-year-olds; n=1,989 female, n=1,578 male)^*^ Source: KiGGS baseline study (2003–2006), KiGGS Wave 2 (2014–2017)

It became apparent that the majority (65.3%) reports sleep problems neither during the KiGGS baseline study nor about ten years later (Figure 5). 14.9% report sleep problems at the first survey point and no longer during young adulthood. A slightly smaller group experienced a worsening: Approximately every eighth participant reports sleep difficulties for the first time in KiGGS Wave 2 (11.8%). 8.0% of the survey participants were affected by sleep problems at both survey points. 43.8% of young women, who reported sleep difficulties as children and adolescents, also had sleep problems as young adults, this number was only 23.1% among young men (total of 34.8%).

As also during the analyses relating to influencing factors, it became apparent that sex plays an important role (Table 3). The group without sleep difficulties at both survey points is smaller among female participants than among male participants (60.1% vs. 70.5%). The opposite applies in the case of the group with sleep problems at both survey points. More than every tenth female participant reported sleep difficulties in the KiGGS baseline study and KiGGS Wave 2 (11.3%). Among young men, this only affected every twentieth (4.6%). Among those, who cross over into the other group, there were also differences between the sexes. Among women, the percentages of those with better and worse sleep quality at the second survey point were identical. Among young men, the group with better sleep during KiGGS Wave 2 was larger.

## 4. Discussion

The results show that sleep difficulties in childhood, adolescence, and young adulthood are common. Almost one quarter of 11- to 17-year-olds reported suffering from difficulties falling asleep or sleeping through the night. In young adulthood (age 18 to 31), sleep problems were also common with a prevalence of almost 20%. Even if no statements can be made as to what extent these are transient phenomena without need for treatment or clinically significant sleep disorders, the results deserve attention. In light of the fact that insufficient sleep is not only relevant for the subjective well-being but is associated with numerous physical and mental adverse effects as well as with limited cognitive functioning and performance, potential for the promotion of health and prevention can be recognised here. However, sleep is currently hardly a topic for public health [32]. While sleep has meanwhile been added as topic into the Healthy People Plan [44] in the United States, which defines the national health promotion goals, there is a lack of a corresponding attention for this topic in Germany.

In the evaluation along sociodemographic features, the significant difference between the sexes is noticeable in both examined groups: Girls and young women reported sleep difficulties significantly more frequently than boys and young men – a pattern, which is confirmed in numerous other surveys (e.g. in DEGS1 [26]). Being a single parent is also associated with a higher prevalence of childhood sleep difficulties. Associations could not be shown for additional sociodemographic variables (age, education of parents, migration background, siblings).

Similar patterns were also reflected in young adulthood, but biographic changes need to also be considered because over one quarter lived with a partner, and over six percent lived with child(ren). While living in a partnership is not relevant in the context at hand, living with – presumably small – children increased the risk for sleep difficulties. The fact that women are affected by this significantly more frequently reflects the still common gendered division of labour in families, according to which women are responsible for taking care of children more frequently [45]. This is also reflected in other health indicators. For instance, women with child(ren) in young adulthood assess their subjective health to be worse than childless women [45].

The regression analyses showed that a plurality of factors influences sleep. For instance, a significant influence of noise on the sleep quality was apparent among the young adults. This supports the considerations of resorting to socioeconomic models, which do not only take into account the individual sleep behaviour but also interpersonal, environmental, and social factors, in promoting healthy sleep [22].

The high significance of health-related variables was also reflected in the regression analyses. Pain conditions verifiably increased the risk for sleep problems, but the importance of limited mental health for the sleep in both groups is particularly striking (among children and adolescents assessed with SDQ, among young adults based on depressive symptoms). The direction of the association, however, is unclear in the analyses at hand. International surveys suggest the conclusion that a bidirectional connection is to be assumed. For instance, Quach et al. [46] show in an Australian longitudinal study that kindergarten-age sleep disorders can lead to internalising (e.g. depression, anxiety) and externalising disorders (e.g. behavioural problems) in later years, and externalising (but not internalising disorders) can result in sleep disorders. Touchette et al. [47] also show that a poor sleep quality at the age of ten is associated with a higher prevalence for externalising problems 20 years later. Mental disorders, however, can also be the cause for sleep disorders, for instance during a treatment with methylphenidate in the case of an attention deficit/hyperactivity disorder (ADHD) [48].

Even though the majority of the respondents did not have any sleep problems at both survey points, at least one third of those, who reported difficulties falling asleep and sleeping through the night as children or adolescents, also indicated sleep problems as young adults. The data at hand does not allow a real longitudinal examination, but international surveys substantiate a high persistence [35]. It is also noteworthy here that young women are more vulnerable: Among them, the percentage of those, who reported sleep problems at both points in time, was twice as large as among young men.

###  

#### Strengths and weaknesses

With KiGGS, there is a representative dataset, which meets high methodological standards and which makes it possible to make differentiated statements. Some limitations must be mentioned nonetheless. For instance, disrupted sleep was only captured with a few items, additionally with different questions at both survey points. The same applies for the influencing factors considered in the analyses. Here, different questions were also asked at different points. Only a first impression of the set of problems can be given with this. Due to the high public health relevance, it is thus advisable to perform a comprehensive survey, which captures sleep (problems) and influencing factors in a differentiated manner. For the systematisation, it lends itself here to plan such a survey on the basis of the socioeconomic models and to additionally take into account the interactions of the influencing factors.

A further limitation is that sleep problems were assessed only from a subjective perspective. The degree of adverse effect caused by the problems was not captured, and a comparison with clinical diagnoses was also not made. Even though the subjective perception of sleep problems is a significant factor influencing well-being, the congruence of subjective assessments and objective findings deserves attention.

With the data at hand, statements can be made at two survey points. It has to remain open thereby, how sleep and sleep problems have developed between these points. Even though international surveys provide indications towards a high persistence of sleep disorders, actual longitudinal statements are not possible with the KiGGS dataset. It also lends itself here to conduct additional surveys in longitudinal perspective.

In summary, it is important to note that it was possible to show first indications of the relevance of sleep problems of children and adolescents by means of the KiGGS data. The high prevalence shows that healthy sleep deserves more attention as public health topic. A mere teaching of sleep-relevant behaviour falls short thereby. On the contrary, interventions must also take into account the social, health, and environmental factors, in order to improve the sleep health of the general public.

## Key statements

- 22.0% of the 11- to 17-year-olds and 19.6% of the 18- to 31-year-olds suffered from sleep problems.
- Sex was an important influencing factor, while other sociodemographic variables were of little importance.
- Pain, noise, and mental problems increased the probability for sleep problems.
- 43.8% of the girls and 23.1% of the boys, who were affected by sleep difficulties, also indicated sleep problems as young adults.

## Figures and Tables

**Table 1:** Prevalence of sleep problems in 11- to 17-year-old girls and boys according to sociodemographic and health variables (n=3,287 girls, n=3,441 boys) Source: KiGGS baseline study (2003–2006)

	Sleep problems					
	Female		Male		Total	
	%	(95% CI)	%	(95% CI)	%	(95% CI)
	**24.9**	**(23.2–26.7)**	**19.2**	**(17.6–20.9)**	**22.0**	**(20.8–23.2)**
**Age group (in years)**						
11**–**13	25.2	(22.8**–**27.7)	22.2	(19.5**–**25.0)	23.6	(21.9**–**25.5)
14**–**17	24.7	(22.4**–**27.1)	17.3	(15.3**–**19.5)	20.9	(19.3**–**22.6)
**Education group**						
Low	28.6	(21.9**–**36.4)	18.2	(13.1**–**24.7)	23.6	(19.3**–**28.6)
Medium	25.2	(22.5**–**28.0)	20.7	(18.3**–**23.4)	22.9	(21.1**–**24.8)
High	23.3	(20.9**–**26.0)	16.6	(14.4**–**19.0)	19.9	(18.3**–**21.6)
**Migration background**						
Without	24.4	(22.3**–**26.7)	18.6	(16.7**–**20.6)	21.5	(20.0**–**23.0)
One-sided	19.9	(14.2**–**27.1)	18.1	(12.5**–**25.5)	19.0	(15.0**–**23.7)
Two-sided	29.2	(25.1**–**33.6)	22.5	(18.7**–**26.7)	25.7	(23.1**–**28.5)
**Type of family**						
With partner in the household^1^	24.2	(22.4**–**26.0)	18.0	(16.4**–**19.7)	21.0	(19.8**–**22.3)
Single parent	29.7	(24.8**–**35.2)	26.3	(21.5**–**31.8)	27.9	(24.3**–**31.8)
**Siblings in the household**						
None	23.9	(20.2**–**28.0)	15.9	(12.9**–**19.5)	19.9	(17.5**–**22.4)
At least one sibling	24.9	(23.0**–**26.8)	19.7	(17.8**–**21.7)	22.2	(20.9**–**23.6)
**Place of residence**						
Rural (<5,000 R)	20.9	(16.6**–**26.0)	16.0	(11.9**–**21.0)	18.3	(15.1**–**22.1)
Provincial (5,000–<20,000 R)	24.0	(21.3**–**27.0)	17.9	(15.2**–**21.0)	20.9	(18.9**–**23.1)
Urban (20,000**–**<100,000 R)	26.0	(22.4**–**29.9)	19.9	(17.0**–**23.2)	22.9	(20.6**–**25.4)
Metropolitan (≥100,000 R)	27.5	(24.7**–**30.5)	22.4	(19.2**–**25.8)	24.9	(23.0**–**26.8)
**Pain in the last three months**						
No	14.1	(10.9**–**17.9)	12.6	(10.4**–**15.2)	13.2	(11.2**–**15.3)
Yes	27.0	(25.1**–**29.1)	21.7	(19.8**–**23.7)	24.5	(23.1**–**25.9)
**SDQ total problem score**						
Normal	20.7	(18.9–22.6)	16.9	(15.3–18.7)	18.7	(17.5–20.0)
Borderline	45.4	(40.3–50.7)	38.2	(31.5–45.4)	42.4	(37.9–46.9)
Abnormal	54.7	(42.6–66.3)	38.3	(25.4–53.0)	47.9	(39.3–56.6)

^1^ incl. with the grandparents, foster parents, placement in a home

CI=confidence interval, R=residents, SDQ=Strengths and Difficulties Questionnaire

**Table 2:** Prevalence of sleep problems among 18- to 31-year-old women and men according to sociodemographic, environmental, and health variables (n=3,384 women, n=2,688 men) Source: KiGGS Wave 2 (2014–2017)

	Sleep problems					
	Female		Male		Total	
	%	(95% CI)	%	(95% CI)	%	(95% CI)
	**26.2**	**(23.9–28.6)**	**13.3**	**(11.5–15.4)**	**19.6**	**(18.1–21.2)**
**Age group (in years)**						
18**–**24	26.2	(23.4**–**29.1)	11.6	(9.7**–**13.9)	18.7	(16.9**–**20.6)
25**–**31	26.2	(22.8**–**29.9)	15.4	(12.3**–**19.1)	20.7	(18.3**–**23.2)
**Education group**						
Low	37.6	(30.7**–**45.1)	12.8	(9.2**–**17.6)	23.5	(19.9**–**27.5)
Medium	26.8	(23.9**–**29.9)	13.5	(11.0**–**16.3)	20.0	(18.0**–**22.2)
High	16.5	(13.1**–**20.6)	11.6	(8.2**–**16.2)	14.2	(11.7**–**17.2)
**Professional status**						
Full-time (incl. military service, voluntary social year)	19.9	(17.3**–**22.8)	13.3	(10.9**–**16.1)	16.1	(14.3**–**18.1)
Part-time (incl. mini job, pupil, student, trainee)	27.5	(23.9**–**31.4)	10.7	(8.5**–**13.5)	19.6	(17.4**–**22.0)
Unpaid work/no employment	45.1	(37.6**–**52.7)	25.0	(16.0**–**36.8)	37.6	(31.8**–**43.8)
(e.g. unemployed, homemaker, parental leave)						
**Migration background**						
Without	24.1	(21.9**–**26.4)	12.8	(10.8**–**15.1)	18.3	(16.7**–**19.9)
One-sided	26.6	(19.4**–**35.3)	16.7	(9.6**–**27.4)	21.3	(15.7**–**28.2)
Two-sided	34.1	(27.5–41.5)	14.0	(9.7**–**19.7)	23.9	(19.9**–**28.3)
**Living with partner**						
Yes	27.1	(23.3**–**31.2)	12.9	(9.5**–**17.2)	21.2	(18.5**–**24.2)
No	25.9	(23.1**–**28.8)	13.5	(11.4**–**16.0)	19.0	(17.2**–**20.9)
**Child(ren) in the household**						
Yes	39.2	(31.6–47.4)	23.7	(13.2**–**38.8)	35.2	(28.7**–**42.3)
No	24.0	(21.7**–**26.4)	12.6	(10.8**–**14.7)	17.9	(16.4**–**19.6)
**Place of residence**						
Rural (<5,000 R)	22.8	(18.8**–**27.4)	13.7	(9.3**–**19.7)	17.9	(14.5**–**22.0)
Provincial (5,000–<20,000 R)	21.0	(16.9**–**25.8)	10.6	(7.8**–**14.2)	15.6	(13.0**–**18.5)
Urban (20,000**–**<100,000 R)	27.6	(23.3**–**32.4)	14.1	(11.1**–**17.8)	20.6	(17.8**–**23.7)
Metropolitan (≥100,000 R)	30.0	(26.1**–**34.2)	14.7	(11.1**–**19.2)	22.5	(19.8**–**25.3)
**Noise exposure**						
Yes	39.3	(33.2**–**45.7)	23.0	(18.0**–**28.8)	31.3	(27.1**–**35.8)
No	22.7	(20.6**–**25.0)	10.7	(8.9**–**12.9)	16.5	(15.0**–**18.1)
**Depressive symptoms (PHQ-9)**						
None (PHQ-9: 0–4)	9.6	(7.7–12.0)	3.6	(2.5–5.3)	6.2	(5.1–7.6)
Mild (PHQ-9: 5–9)	28.9	(25.2–33.0)	18.1	(14.3–22.7)	23.8	(21.1–26.7)
Moderate to severe (PHQ-9: 10–27)	58.6	(53.0–63.9)	39.9	(32.3–48.0)	50.6	(46.2–55.0)

CI=confidence interval, R=residents, PHQ-9=Patient Health Questionnaire

**Table 3:** Prevalence of sleep problems at two survey points – transitions (11- to 17-year-olds and 21- to 31-year-olds; n=1,989 female, n=1,578 male) Source: KiGGS baseline study (2003–2006), KiGGS Wave 2 (2014–2017)

Sleep problems in the KiGGS baseline study	Sleep problems in KiGGS Wave 2	Female		Male		Total	
		%	(95% CI)	%	(95% CI)	%	(95% CI)
Yes	Yes	11.3	(9.5–13.1)	4.6	(3.2–6.0)	8.0	(6.8–9.2)
Yes	No	14.5	(12.6–16.5)	15.3	(13.0–17.7)	14.9	(13.3–16.5)
No	Yes	14.1	(12.0–16.2)	9.6	(7.9–11.3)	11.8	(10.4–13.2)
No	No	60.1	(57.3–63.0)	70.5	(67.6–73.3)	65.3	(63.2–67.3)

CI = confidence interval
